# Percutaneous transhepatic intrahepatic portosystemic shunt for variceal bleeding: A series of six cases and literature review

**DOI:** 10.1016/j.jimed.2020.10.007

**Published:** 2020-10-12

**Authors:** Hang Du, Binyan Zhong, Peng Zhang, Wansheng Wang, Jian Shen, Shuai Zhang, Wanci Li, Haohuan Tang, Linfeng Zhou, Weihao Yang, Xiaoli Zhu

**Affiliations:** a Department of Interventional Radiology, The First Affiliated Hospital of Soochow University, Suzhou, China; b Department of Interventional Radiology, Hubei Cancer Hospital, Hubei Cancer Research Institute, Affiliated Cancer Hospital of Tongji Medical College, Huazhong University of Science and Technology, HuBei, China

**Keywords:** Transjugular intrahepatic portosystemic shunt, Percutaneous transhepatic intrahepatic portosystemic shunt, Esophageal and gastric varices

## Abstract

**Objectives:**

To present a case series of modified transjugular intrahepatic portosystemic shunts (TIPS) and percutaneous transhepatic intrahepatic portosystemic shunts (PTIPS) in cirrhotic patients with variceal bleeding (VB). In addition, the scientific literature pertaining to PTIPS was reviewed.

**Methods:**

This retrospective clinical case series included six cirrhotic patients with VB who were treated with PTIPS after the failure of endoscopic band ligation or endoscopic injection sclerotherapy combined with vasoactive drugs. The treatment was conducted between January 2017 and June 2019 at a single institution. Three patients suffered from severe atrophy of the right or left lobar of the liver as well as the main right or left branch of the portal vein. The remaining three patients showed severe atrophy of the whole liver and portal vein, resulting in widening of the liver fissure. A paired *t*-test was used to compare the changes in portal pressure gradient between before and after the PTIPS operation. The rebleeding rate, treatment efficacy, complications, and technical success rate were all assessed during follow-up.

**Results:**

All six PTIPS procedures were performed successfully, with no severe procedural-related complications observed. None of the patients experienced VB during a mean follow-up of 22.8 (range, 18.0–28.0) months. The mean portosystemic pressure gradient decreased from 28.3 ​± ​4.3 ​mmHg pre-procedure to 12.3 ​± ​2.6 ​mmHg immediately post-procedure (P ​< ​0.001). At follow-up, one patient was found to have developed grade 2 hepatic encephalopathy thrice during the first year, according to the West Haven criteria. However, this was resolved following medical treatment.

**Conclusions:**

When the patient’s portal venous anatomy is unconducive to the performance of TIPS using the transjugular approach, PTIPS can be considered as a safe, effective complementary surgical approach for patients with VB.

## 1 Introduction

Variceal bleeding (VB) is a life-threatening complication for patients with cirrhosis and portal hypertension. The recommended first-line treatment for VB is endoscopic band ligation (EBL) in conjunction with vasoactive drugs and nonselective beta-blockers.^1^ In the event that first-line treatment fails, transjugular intrahepatic portosystemic shunt (TIPS) is recommended as a second-line choice.^1^^,^^2^

TIPS is an imaging-guided procedure that involves connecting the portal and hepatic veins in the liver using a transjugular approach.^3^^,^^4^ Nevertheless, in cases with atrophy of both the liver and portal vein or atrophy of the liver with broadened liver fissure and a slim portal vein, antegrade puncture from the hepatic vein to the portal vein becomes difficult and even dangerous. As surgical techniques have improved with time, a modified TIPS technique, referred to as percutaneous transhepatic intrahepatic portosystemic shunt (PTIPS), has emerged, involving the use of stents for cases with challenging anatomy^5^^,^^6^^,^.^7^ However, the complexity and increased operating time of this procedure is accompanied by an increased risk of surgical complications. The aim of the procedure is to create a portal-systemic connection by puncturing the portal vein into the hepatic vein or inferior vena cava (IVC). This study reports six cases of cirrhotic patients with distinctive vascular anatomy who underwent PTIPS for the treatment of VB.

## 2 Methods

### 2.1 Ethical approval

The Institutional Review Board of the First Affiliated Hospital of Soochow University approved this study. All clinical practices and observations were conducted in accordance with the Declaration of Helsinki. Informed consent was obtained from each patient before the study was conducted.

### 2.2 Patient characteristics

We included six cirrhotic patients with portal hypertension and VB who were treated with PTIPS at The First Affiliated Hospital of Soochow University between January 2017 and June 2019. All patients had either refractory VB or a history of failed endoscopic and medical treatment. In addition, they presented with distinct vascular anatomy of the puncture pathway during their pretreatment assessments, leading their respective physicians to the decision that TIPS would be too difficult to perform. The baseline characteristics of the patients are presented in greater detail in Table 1.

**Table 1:** Clinical characteristics before PTIPS.

NO	Gender age	CTP	MELD score	Ascites	Varices features	History of treatments	Distinctiveness	Follow-up (months)
1	M/48y	C	17	+	GOV1	HCC-Right hepatectomy/EBL/octreotide/NSBBs	The right hepatic lobe and right portal vein atrophied after Right hepatectomy	18
2	M/54y	B	13	/	GOV1	Splenectomy/EIS/octreotide/NSBBs	Atrophied liver with broadened liver fissure, and finespun portal vein	21
3	M/63y	A	8	/	GOV1	Splenectomy/HCC-Right hepatectomy/TACE/octreotide/NSBBs	The right hepatic lobe and right portal vein atrophied after Right hepatectomy combined TACE therapy	21
4	M/55y	A	9	/	GOV1	HCC-Left hepatectomy/TACE/octreotide/NSBBs	The left hepatic lobe and left portal vein atrophied after hepatectomy and TACE therapy, and with finespun portal vein	24
5	F/51y	C	16	/	GOV1	Splenectomy/EIS/octreotide/NSBBs	Chronic portal vein thrombosis after splenectomy; Atrophied liver with broadened liver fissure ​and finespun portal vein	25
6	M/65y	C	14	/	GOV1	TACE/RFA/EBL/octreotide/NSBBs/PTVE	The right hepatic lobe and right portal vein atrophied severely after TACE and RFA	28

CTP = Child-Turcotte-Pugh. MELD ​= ​Model for End-stage Liver Disease. GOV ​= ​gastro-esophageal varices. HCC ​= ​hepatocellular carcinoma. NSBBs ​= ​Non-selective β-blockers. EBL ​= ​endoscopic band ligation. EIS ​= ​endoscopic injection sclerotherapy. TACE ​= ​transarterial chemoembolization. RFA ​= ​radiofrequency ablation. PTVE ​= ​percutaneous transhepatic variceal embolization.

### 2.3 PTIPS procedure

**Fig. 1:**
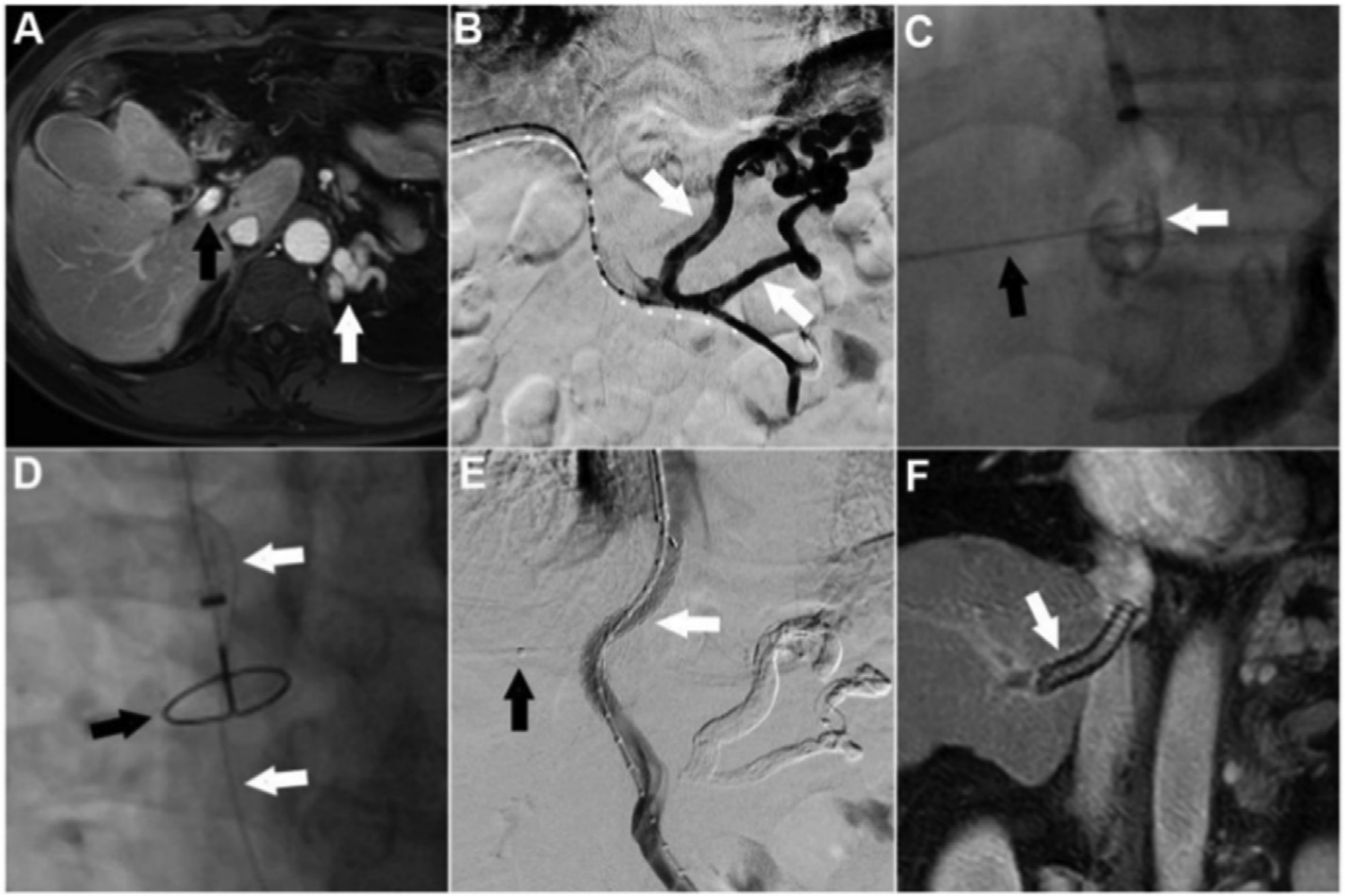
Number four patient: A) The patient showed severe atrophy of the left hepatic lobe and left portal vein after hepatectomy and TACE therapy, and the portal vein completely exposed outside the liver parenchyma (black arrow). B) Two gastric varices (white arrow) with gastrorenal shunts was confirmed by transhepatic portography. C) A 21-gauge, 15-cm needle (black arrow) was used to puncture the gold-marked catheter (white arrow) via a 6-Fr sheath in the right portal vein. D) Transhepatic puncture from right portal vein to IVC was performed successfully and the exchange guidewire (white arrow) was captured by a snare (black arrow). E) A Viatorr stent (8 ​× ​60 mm, 20 mm) was inserted and post-stent venography was performed. The 6F catheter (black arrow) was removed after the transhepatic puncture tract was embolized with 14mm-8cm coils. F) MRI scan 24 months after TIPS.

All PTIPS procedures were performed under local anesthesia and sedation by two interventional radiologists (Fig. 1). Under ultrasonic guidance, the right portal vein was accessed with a 21-gauge, 15-cm needle (Cook Medical, Bloomington, IN, USA) via a percutaneous transhepatic approach, after which a 6-Fr sheath (Terumo Corporation, Tokyo, Japan) was placed. A 5-Fr pigtail angiography catheter (Cook Medical, Bloomington, IN, USA) was inserted into the main portal vein under the assistance of a guidewire, and venography was applied to confirm the type and degree of the varices. Subsequently, the portal pressure was measured and recorded (Fig. 1B). The gastrorenal shunts were embolized with coils (Cook Medical, Bloomington, IN, USA) and *N*-butyl-2-cyanoacrylate (Compont Medical Devices Co, Beijing, China) via a coil-assisted retrograde transvenous obliteration approach.

A 9-Fr sheath (RUPS-100; Cook Medical, Bloomington, IN, USA) was used and placed into the inferior vena cava (IVC) via the right internal jugular vein. A gold-marked catheter (Cook Medical, Bloomington, IN, USA) was placed in the hepatic segment of the IVC. Venography and systemic pressure measurement were then performed. In addition, the pre-TIPS portosystemic pressure gradient (PPG) was calculated. A 21-gauge, 20-cm percutaneous transhepatic cholangiography needle (external diameter ​= ​0.82 mm) was inserted through a 6-Fr sheath to puncture the gold-marked catheter of the IVC (Fig. 1C). After successful puncture to the IVC, which was confirmed by aspirated blood and injected contrast medium, a 0.018-inch guidewire (Terumo Corporation, Tokyo, Japan) was inserted into the right atrium. The 0.018-inch guidewire was exchanged with a 0.035-inch guidewire (Terumo Corporation, Tokyo, Japan) after introducing a 4-Fr catheter. This wire was then captured by a snare (Amplatz GooseNeck® Snare Kit, EV3, USA) and pulled through the transjugular sheath (Fig. 1D).

An 8 ​× ​6-mm, 130-cm balloon catheter (Boston Scientific, Watertown, MA, USA) was advanced transjugularly in order to dilate the space between the IVC and the puncture point in the portal vein. Stent insertion was then performed using standard techniques. An 8 mm expanded polytetrafluoroethylene-covered stent (Viatorr®; W.L. Gore and Associates, Flagstaff, AZ, USA) was inserted. Transjugular portography confirmed the patency of the shunt tract (Fig. 1E). Subsequently, post-TIPS PPG was measured. The transhepatic puncture tract was embolized with 14 mm–8 cm coils (Cook Medical, Bloomington, IN, USA), and the 6-Fr catheter was removed. Finally, the transjugularly-inserted 9-Fr sheath was removed.

### 2.4 Post-procedure assessments and follow-up

Postoperatively, all patients underwent anticoagulation with low-molecular-weight heparin for 3–5 days. Rivaroxaban was administered orally for 6 months to 1 year. All patients were followed up at 1, 3, and 6 months, and every 6 months thereafter. Blood and coagulation function tests were performed at every follow-up visit. Color Doppler ultrasonography evaluation was performed at 1, 3, and 6 months, and every 6 months thereafter, in order to assess shunt patency and hemodynamic changes. Computed tomography (CT) or endoscopy was performed if necessary. The patients were admitted to our department once they had recurrent bleeding, hepatic encephalopathy, or other severe complications. The follow-up period was defined as the time interval between TIPS surgery and liver transplantation, death, or the last follow-up visit (June 30, 2019).

## 3 Results

Of the six patients, three suffered from severe atrophy of the right or left lobar of the liver and main right or left branch of the portal vein due to prior hepatic resection or interventional treatment. The other three patients had severe atrophy of the whole liver and the tenuous portal vein, which resulted in widening of the liver fissure and complete exposure of the bifurcation of the portal vein outside the liver parenchyma. All of the patients had previously undergone failed endoscopic and medical treatment. The PTIPS procedures were performed successfully within 72 hours of admission without severe procedure-related complications, such as intraperitoneal hemorrhage, perforation of the liver capsule, rupture of the external hepatic portal vein, or biliary fistula. The mean PPG decreased from 28.3 ​± ​4.3 ​mmHg pre-procedure to 12.3 ​± ​2.6 ​mmHg immediately post-procedure (P ​< ​0.001). During a mean follow-up of 22.8 (range, 18.0–28.0) months, one patient developed grade 2 hepatic encephalopathy thrice within the first year, according to the West Haven criteria.^8^ This was resolved following medical treatment. None of the patients experienced variceal rebleeding.

### 3.1 Case presentation

**Fig. 2:**
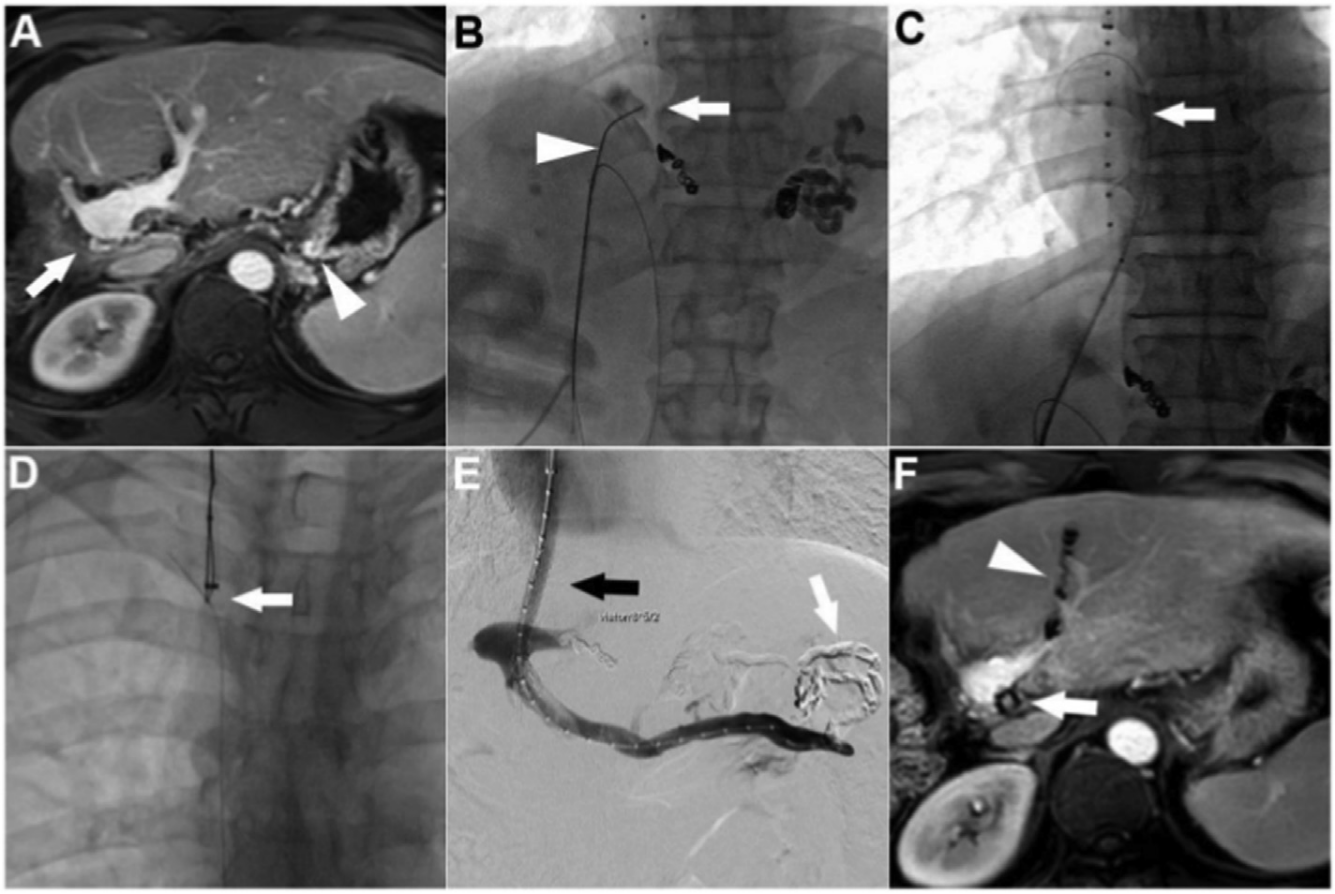
Number six patient: A) The patient suffered from severe atrophy of the right hepatic lobe and right portal vein (arrow) after TACE combined with RFA therapy, and varices were founded in the fundus of the stomach (triangle). B) A 21-gauge, 15-cm needle (triangle) was used to puncture the gold-marked catheter (arrow) via a 6-Fr sheath. C) The 0.035-inch guidewire (arrow) was exchanged into the right atrium. D) The guidewire was captured by a snare (arrow) through the transjugular sheath. E) Viatorr stent was inserted (black arrow) after variceal embolization (white arrow). F) Transhepatic puncture tract was embolized with coils (triangle), imaging follow-up at 24 months identified that the stent was patent (arrow) and no recurrent malignant lesion were observed.

A 65-year-old man was referred to our department because of acute variceal bleeding. He underwent radiofrequency ablation combined with transarterial chemoembolization for the treatment of hepatocellular carcinoma 33 months prior. Complete tumor response was achieved after the combination treatment, although the right lobar of the liver and main right branch of the portal vein became severely atrophied (Fig. 2A). PTIPS was performed for the treatment of variceal bleeding after a combination of endoscopy, medical treatment, and percutaneous transhepatic variceal embolization had failed to elicit improvements. Under ultrasonic guidance, the left sagittal portal vein was accessed with a 21-gauge, 15-cm needle via a percutaneous transhepatic approach. A 6-Fr sheath was then placed after successful puncture of the left portal vein, and a 0.035-inch guidewire was advanced to the superior mesenteric vein as a safety wire. According to the imaging data, the tip of the puncture needle was angled by approximately 40° (Fig. 2B). After successful puncture of the pigtail catheter, the 6-Fr sheath was then advanced into the IVC under the guidance of a 0.018-inch guidewire. Thereafter, the 0.035-inch guidewire was exchanged in the right atrium (Fig. 2C), captured by a snare, and pulled through the transjugular sheath (Fig. 2D). Portal venography and PPG measurements were performed before the variceal embolization. Balloon dilatation was performed using an 8 ​× ​6-mm, 130-cm balloon catheter, after which a Viatorr® stent (8 ​× ​50 mm, 20 mm) was inserted (Fig. 2E). PPG decreased from 23.3 ​mmHg pre-procedure to 10.7 ​mmHg immediately post-procedure. Imaging follow-up at 24 months revealed that the stent was patent, and no recurrent malignant lesions were observed (Fig. 2F). No variceal rebleeding was observed during follow-up.

## 4 Discussion

The standard TIPS is an imaging-guided procedure for connecting the hepatic and portal veins in the liver using a transjugular approach. Nevertheless, TIPS is difficult to perform when the vascular anatomy of the hepatic or portal vein is uncommon.^9^ One of the most frequently-used variations of TIPS is direct intrahepatic portacaval shunt (DIPS), which changes the connection from portal-hepatic to portal-caval^9^^,^^10^^,^.^11^ It is mainly used for Budd-Chiari syndrome and multiple occlusions of the hepatic vein.^10^^,^^12^ DIPS is performed using a transjugular approach under the assumption that the retrohepatic segment of the IVC is surrounded by the caudate lobe of the liver. In addition, the puncture site of the portal vein should not be completely exposed outside the liver parenchyma.^13^

Patients with severe cirrhosis often suffer from a severely atrophied liver, and the puncture site of the portal vein is completely exposed outside the liver parenchyma. In this condition, transhepatic venous puncture of the portal vein can lead to puncture of the main portal vein or celiac artery by mistake, increasing the risk of rapid bleeding.^14^ The portal puncture site of the patients reported in this case series was completely exposed outside the liver parenchyma, which made it difficult to perform TIPS or DIPS using a transjugular approach. Another modification of the TIPS technique, namely percutaneous transhepatic intrahepatic portosystemic shunt, is a better choice in patients with this condition. Multiple image guidance techniques such as fluoroscopy (landmarks, direct/indirect/wedged portography, or CO2), three-dimensional CT, ultrasound (US), or intravascular ultrasonography (IVUS), have all been adopted to facilitate portal vein access in patients with challenging anatomy.^14^^,^^15^^,^^16^^,^^17^ According to our clinical experience, US combined with fluoroscopic guidance is the most convenient method for retrograde puncture of the hepatic segment of the IVC following safe percutaneous transhepatic puncture.

Aytekin et al.^5^ applied this type of portacaval shunt using the percutaneous transhepatic transjugular technique in patients with occluded or small hepatic veins, as did Chen et al.^18^ The creation of this shunt requires a sharply curved needle owing to the posterior anatomical position of the IVC as compared with the hepatic vein. To solve this problem, Aytekin et al. inserted two snares, one in the portal vein and one in the IVC. They then rotated the image intensifier in order to achieve right oblique projection with caudal angulation, thereby superimposing the snares. After successful placement, an 18-gauge, 15-cm Chiba needle was advanced directly through the two snares under fluoroscopic guidance. Chen et al. used a 20-gauge Chiba needle with a tip that was angled 30–40° in the most distal centimeter to puncture the hepatic vein via the portal vein, using the hepatic vein catheter as a marker. Unlike previous reports, we angled the 21-gauge, 15-cm needle based on the findings of the enhanced CT images and fluoroscopic angiography.

This brief report found PTIPS to be a safe and effective approach for the treatment of variceal bleeding in cirrhotic patients with distinct vascular anatomy. However, future studies using large sample sizes and long-term follow-up are needed to confirm these findings.

## Financial support

This study was supported by the 10.13039/501100013059Jiangsu Provincial Medical Talent Funding (ZDRCA2016038), the Suzhou Special Diagnosis and Treatment Technology of Clinical Key Diseases (LCZX201704), the 10.13039/501100001809National Natural Science Foundation of China (81771945, 81901847), the 10.13039/501100004608Natural Science Foundation of Jiangsu Province (BK20190177) and the Suzhou Science and Technology Youth Plan (KJXW2018003).

## Patient consent

Witten informed consent was obtained from patients for publication of these case reports and any accompanying images.

## Declaration of competing interest

The authors declared that they have no conflicts of interest to this work. We declare that we do not have any commercial or associative interest that represents a conflict of interest in connection with the work submitted.
